# Fragment Reattachment after Atypical Crown Fracture in Maxillary Central Incisor

**DOI:** 10.1155/2014/231603

**Published:** 2014-12-24

**Authors:** Vanessa Torraca Peraro Vaz, Cristina Dupim Presoto, Keren Cristina Fagundes Jordão, André Gustavo Paleari, Andrea Abi-Rached Dantas, José Claudio Martins Segalla, Osmir Batista de Oliveira Junior

**Affiliations:** ^1^Department of Restorative Dentistry, Araraquara Dental School, Univ. Estadual Paulista (UNESP), Rua Humaitá 1680, 14801-903 Araraquara, SP, Brazil; ^2^Department of Dental Materials and Prosthodontics, Araraquara Dental School, Univ. Estadual Paulista (UNESP), Rua Humaitá 1680, 14801-903 Araraquara, SP, Brazil

## Abstract

*Background*. Fracture by trauma is one of the most common types of dental injury in the permanent dentition among children and teenagers. *Aim*. The aim of this study was to report the treatment performed to an atypical dental trauma case in a maxillary central incisor of a young patient by means of reattachment of the tooth fragment. *Case Description*. A 12-year-old male patient suffered a vertical crown fracture to the maxillary right central incisor. After clinical and radiographic examinations, a conservative restorative treatment which consisted in the reattachment of the tooth fragment with flow resin was performed in order to preserve the dental element and to obtain maximum aesthetics. *Conclusion*. The reattachment of fractured fragment is a fast and easy technique that can be used successfully as an option to restore dental element which suffered trauma. *Clinical Significance*. This technique restores the aesthetics and function of the dental element with minimal discomfort to the patient.

## 1. Introduction

Children and adolescents are the majority of patients with dental trauma history and the maxillary central incisors are the most affected teeth, due to their position at the arch and to a large incisal overjet [1–5].

Traditionally, the causes of dental injuries are sports falls, aggressions, and accidents [6–8]. Sanbuncuoglu [9] suggests that attention deficit disorder and hyperactivity may aggravate such situations, increasing the individual predisposition to dental traumas. According to Brown et al. [10], the hyperactivity affects 4–12% of school-age children.

Several techniques have been advocated for the restoration of fractured teeth, such as resin, ceramic or steel crowns, orthodontic bands, and resin composite restorations with and without pins [6–11]. In view of the treatment modalities for no complex crown fractures, fragment reattachment might be considered an acceptable option to restore traumatized dental elements [4, 6, 11].

This paper reports an atypical dental trauma case in a maxillary central incisor of a young patient with crown fracture. A conservative restorative treatment which consisted in the reattachment of the tooth fragment was performed in order to preserve the dental element and to obtain maximum aesthetics.

## 2. Case Presentation

A 12-year-old male patient, with a history of hyperactivity, was referred to Restorative Dentistry Clinic of Paulista Association of Dental Surgeons, Araraquara, Brazil, with a crown fracture to the maxillary right incisor tooth that occurred while practicing sports one day ago.

**Figure 1:**
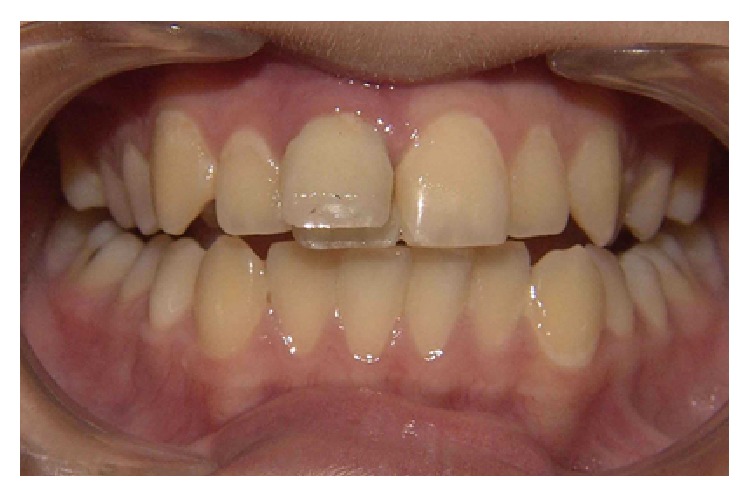
Clinical aspect of the crow fracture to the maxillary right central incisor.

**Figure 2:**
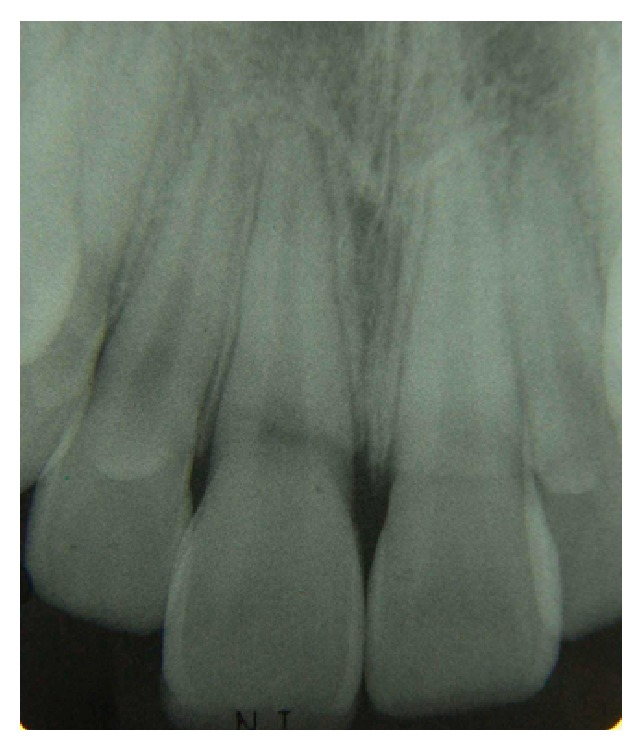
Initial periapical radiograph.

Intraoral clinical examinations (Figure 1) showed a vertical crown fracture to the maxillary right central incisor where the fracture line passes mesiodistally through the crown and extended to cervical direction with pulp exposure. It does not find any associated root fracture after intraoral periapical radiograph (Figure 2).

The chosen treatment was a less invasive technique, which consisted in the reattachment of the tooth fragment, since it is an immediate restorative technique, does not involve surgical procedures or anesthesia, reduces the possibility of gingival recession, and promotes an immediate repair of the aesthetics and function of the fractured tooth.

**Figure 3:**
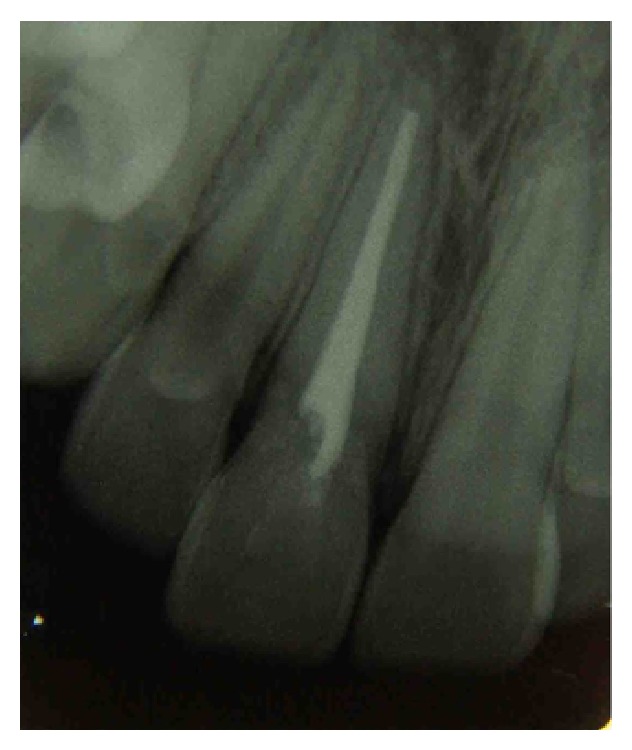
Radiograph after endodontic treatment.

After approval of the proposed treatment, the endodontic treatment was performed due to the wide exposure of the dental pulp. Manual instrumentation was performed by the step-back technique with the aid of Gates Glidden drills #2, #3, and #4. The root canal was then obturated by the classical technique with lateral condensation (Figure 3).

**Figure 4:**
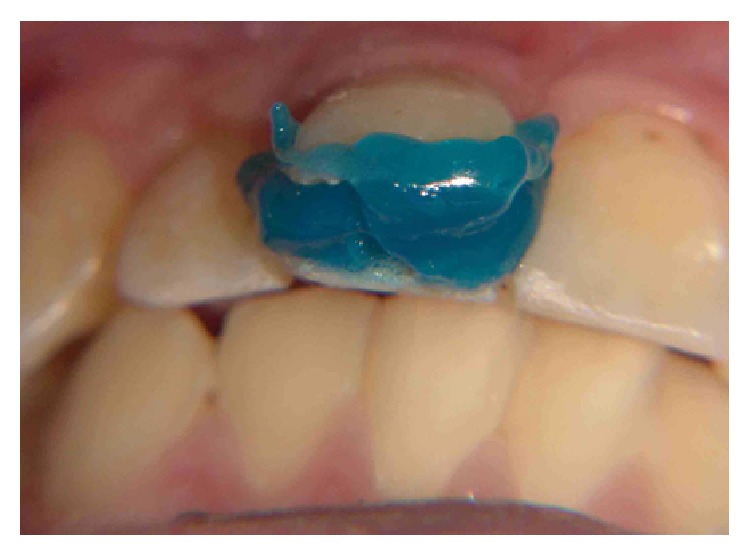
Acid etching on enamel and dentin.

**Figure 5:**
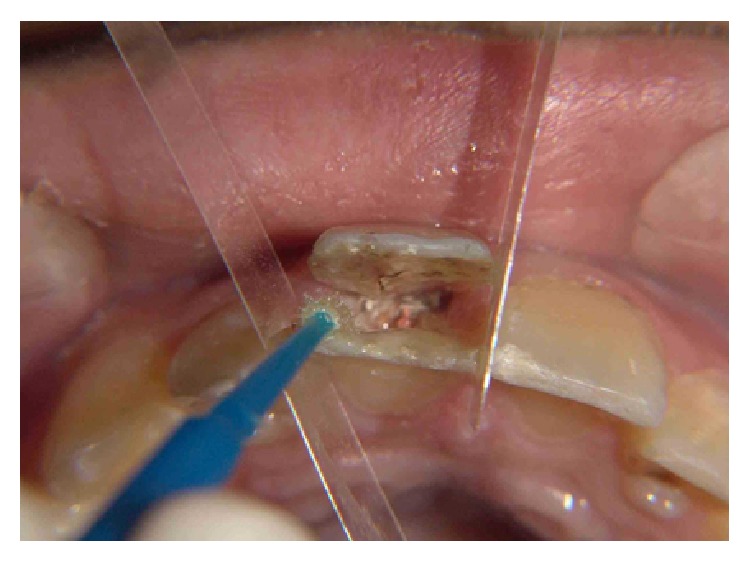
Application of adhesive system.

**Figure 6:**
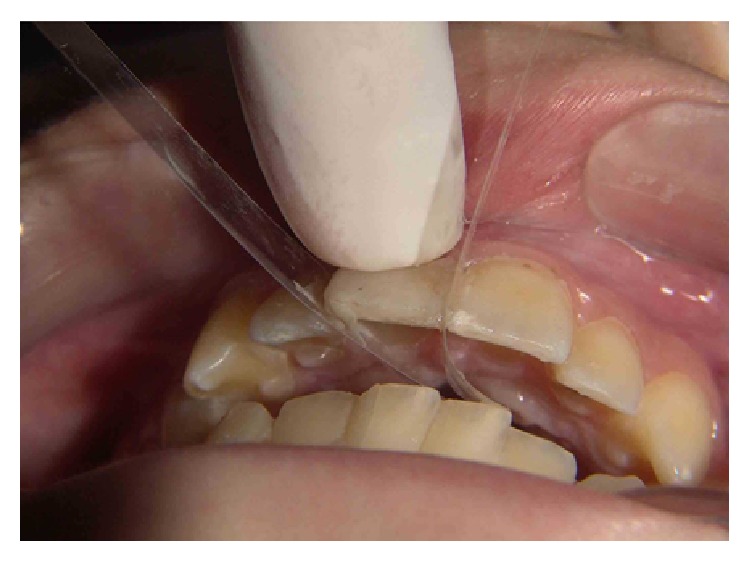
Reattachment of the tooth fragment with a flow resin.

The cavity was decontaminated with 0.2% chlorhexidine previous to 37% phosphoric acid (Acid Gel, Villevie, Joinville, SC, Brazil) etching on enamel margins (30 seconds) and subsequent dentin (15 seconds) (Figure 4). The acid-conditioned surface was rinsed thoroughly in order to remove the acid. Excess of water was removed and the dentin surface was dried with absorbent paper. The primer (Scotchbond Multi-Purpose Plus, 3M ESPE, St. Paul, MN, USA) was actively applied in 2 layers and dried for five seconds with air stream. Then, the adhesive (Scotchbond Multi-Purpose Plus, 3M ESPE, St. Paul, MN, USA) layer was applied, respecting the recommendations of the manufacturer (Figure 5). A polyester matrix was fitted to protect the adjacent tooth and a flow resin (Filtek Z350 XT 3M ESPE, St. Paul, MN, USA) was used to reattach the tooth fragment (Figure 6). A prepolymerization was performed and the remaining resin was removed with a Hollemback instrument.

**Figure 7:**
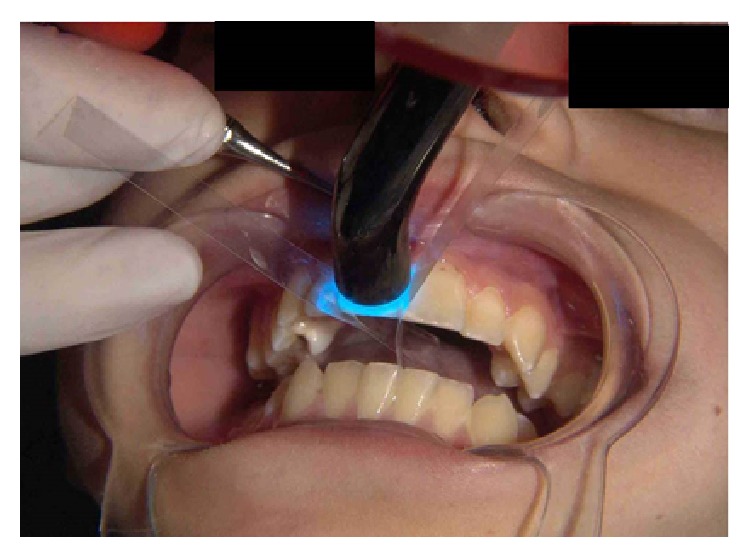
Photopolymerization of the flow resin.

While the fragment was lightly pressed, the dental floss was used to clean the proximal surfaces from the restorative material. Afterwards the fragment was light cured and the resin polymerization was completed (Figure 7).

**Figure 8:**
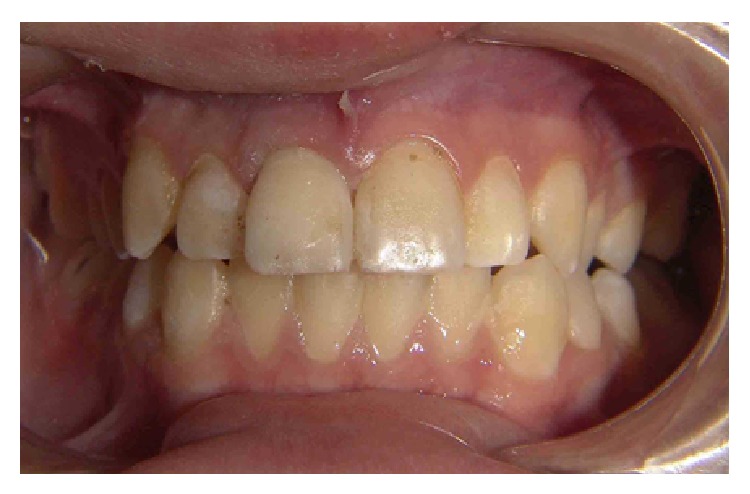
Clinical appearance after the fragment reattachment.

The fracture line was polished with rubber abrasive at low speed to avoid or minimize the biofilm accumulation. The occlusion was checked using a carbon in the palatal surface of the restored element and the patient was asked to bite in maximum intercuspal position and to make protrusion movement (Figure 8).

The patient returned to the clinic after 48 hours for another occlusal adjustment and marginal polishing. Periodic returns (each 6 months) for the dental element preservation were recommended. The patient was conducted to psychological accompanying due to the mother's report about the signs of hyperactivity and attention deficit disorder.

## 3. Discussion

Dental trauma can be considered one of the most serious problems that affect the oral health of children and adolescents. Fractures of the anterior teeth affect function and dental aesthetics and promote emotional problems for patients such as social isolation, depression, and feed abstinence, among other factors [1].

The main causes of trauma that affect the permanent incisors are falls, collisions, sports, violence (fights), and car accidents [2, 3]. Central incisors present an increased overjet in children which may promote a predisposition to trauma [3]. Another aggravation of dental trauma is the hyperactivity/attention deficit disorder, a condition that affects 4–12% of school-age children, causes anxiety and attention disorders, and may increase the incidence of traumas [10].

The hyperactive patient can disturb the restorative procedure since the patient cannot keep quiet on the dental chair and control his tongue movements. The clinician must be aware of such difficulties while deciding which restorative technique is the most appropriate for dental trauma.

The type of fracture is decisive at the moment of restorative technique selection. According to DiAngelis et al. [12], coronary fracture is the simplest fracture and may be made with resin flow which enables the resin sealing and avoids its opening. The most complicated tooth fracture is the root fracture and the element loss may be the most likely treatment.

This case report is an unusual example of coronal fracture, of which the upper left central incisor fractured in the mesiodistal direction separating buccal and lingual surfaces and exposed the fracture line at the histological sulcus. The conventional treatment would be gingivectomy or orthodontic traction, to expose the fracture line and to avoid the contamination by gingival fluid, facilitating its maintenance by the patient [13]. Depending on the specific feature of this case (the periodontal fibers from the buccal fragment remained properly connected with no bleeding even after trauma), the direct reattachment was the chosen treatment, because it presented many advantages as no need of orthodontic treatment, optimal functional and aesthetic results obtained, and minimal discomfort caused for the patient [14, 15]. The direct reattachment of the fragments was also performed due to the great innovation of new adhesive systems [5] and also because the patient presented signs of hyperactivity, immaturity, and anxiety.

## 4. Conclusion

The reattachment of fractured fragment is a fast and easy technique that can be used successfully as an option to restore dental element which suffered trauma.
